# Ectopic Cushing's Syndrome Secondary to Metastatic Paraganglioma

**DOI:** 10.1155/2021/5593920

**Published:** 2021-06-24

**Authors:** R. Daya, C. Wingfield, P. Sotshononda, F. Seedat, S. Bulbulia, M. D. Simmons, M. Louw, Z. Bayat

**Affiliations:** ^1^Division of Endocrinology and Metabolism, Department of Internal Medicine, Helen Joseph Hospital, 1 Perth Road, Rossmore, Johannesburg, South Africa; ^2^Division of Endocrinology and Metabolism, Department of Internal Medicine, Faculty of Health Sciences, School of Clinical Medicine, University of the Witwatersrand, Johannesburg, South Africa; ^3^Department of Anatomical Pathology, School of Pathology, University of the Witwatersrand, Johannesburg, South Africa

## Abstract

Paraneoplastic or ectopic Cushing's syndrome (CS) is a rare cause of endogenous hypercortisolism. It is due to ectopic adrenocorticotropic hormone (ACTH) secretion and has been reported in association with a variety of neuroendocrine tumors such as small-cell lung carcinoma, carcinoid tumors, and medullary carcinoma of the thyroid. Paragangliomas (PGLs) are rare neuroendocrine tumors that can secrete catecholamines. Case reports and reports of ectopic ACTH secretion from metastatic PGLs causing CS are exceedingly rare. We present a case of a 38-year-old female, who presented with typical signs, symptoms, and complications of CS, secondary to a PGL with widespread metastases, which eventually led to her demise.

## 1. Introduction

Pheochromocytomas (PCCs) and paragangliomas (PGLs) are rare neuroendocrine tumors, collectively termed pheochromocytoma–paraganglioma (PPGL). PCCs usually arise from the adrenal medulla, whilst PGLs arise from chromaffin tissue outside of the adrenal medulla and can secrete neuropeptides and catecholamines so are also referred to as extra-adrenal PCC.

Cushing's syndrome (CS) is a collection of signs and symptoms due to prolonged exposure to either endogenous or exogenous excess glucocorticoids (GCs) resulting in a disruption of the normal hypothalamic-pituitary-adrenal axis [1]. CS is broadly categorised as adrenocorticotropic hormone (ACTH) dependent (70–80%) or ACTH independent (20–30%) [2]. ACTH-dependent CS occurs most commonly secondary to a pituitary corticotrophic adenoma (Cushing's disease), whilst less frequently, it may be caused by an extrapituitary tumor (ectopic ACTH secretion) or rarely a tumor secreting corticotropin-releasing hormone (CRH) [2].

Ectopic CS is a rare cause of endogenous hypercortisolism accounting for 5–20% of all CS cases and 10–20% of ACTH-dependent CS patients [2]. It is due to ectopic ACTH secretion and has been reported in association with a variety of neuroendocrine tumors such as small-cell lung carcinoma, carcinoid tumors, and medullary carcinoma of the thyroid. Ectopic ACTH secretion from PGLs is rare and limited to case reports. Case reports and reports of metastatic PGL causing CS are exceedingly rare.

## 2. Case

A 38-year-old female presented to hospital with a long-standing history of progressive abdominal pain and distension. Functional inquiry revealed complaints of amenorrhoea, worsening malaise, proximal muscle weakness, insomnia, weight gain, and persecutory delusions.

She had a background history of Human Immunodeficiency Virus (HIV) infection, for which she was on second-line antiretroviral therapy (ARV) (tenofovir, lamivudine, and dolutegravir) due to previous virological failure, with no history of any prior opportunistic infections. In addition, she was diagnosed with hypertension and type 2 diabetes mellitus a few years prior, which was insufficiently controlled (glycated haemoglobin (HbA1 c) level of 7.6%) on admission. She had no micro- or macrovascular complications. Her medication included amlodipine and metformin; however, she was not on any medication for her mental health complaints. There was no history of headaches, palpitations, or hyperhidrosis. She denied any symptoms of sleep apnoea. She was a nonsmoker and denied any history of exogenous corticosteroid use (oral, nasal, topical, or injectable). There was no family history of Cushing's syndrome/disease or pheochromocytoma.

Her body weight and height were 183 pounds (83 kg) and 5 foot 4 inches (165 cm), respectively, with a body mass index of 30.5 kg/m^2^. Physical examination revealed typical cushingoid features including moon facies, facial plethora, and prominent truncal, facial, and nuchal adiposity, as well as multiple areas of bruising. Abdominal distention was noted, with visible violaceous striae and no palpable masses. The rest of the clinical examination was normal.

Her latest CD4 lymphocyte count was 131 cells/mm^3^ with an undetectable viral load. Fasting blood glucose levels were elevated. Biochemical investigations revealed an elevated random serum cortisol level of 1118 nmol/L (133–537 nmol/L), with an inappropriately normal adrenocorticotrophic hormone (ACTH) level of 4.6 pmol/L (1.6–13.9 pmol/L) (Table 1). In addition, there was an elevated 24-hour urinary free cortisol level of 10447 nmol/24 hours (10–124 nmol/24 hours). A repeat sample confirmed an elevated urinary free cortisol level. Low- (1 mg) and high-dose (8 mg) dexamethasone suppression tests were conducted, and both showed an inability to suppress plasma cortisol levels (Table 2). The rest of the biochemical investigations are shown in Table 1.

Based on her history, clinical examination, and investigations, a diagnosis of CS was made. To determine the underlying etiology, further imaging was carried out.

**Figure 1:**
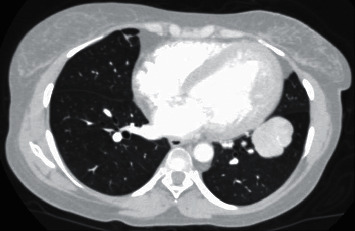
Contrast-enhanced computed tomography (CT) of the chest showing a lung mass measuring 4.4 × 4.0 × 4.0 cm.

**Figure 2:**
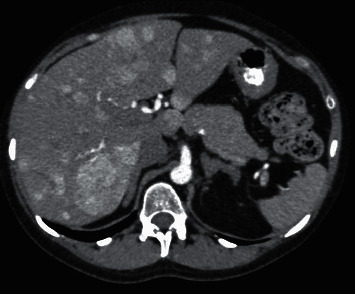
CT of the abdomen showing multiple irregular lesions in the liver, with the largest measuring 6.0 × 6.0 cm (white arrow).

Abdominal sonography showed hepatomegaly (span of 188 mm) with multiple hypodense hepatic lesions suggestive of metastatic disease. The adrenal glands, pancreas, spleen, and kidneys were normal. In addition, the pituitary gland was normal on imaging. Contrast-enhanced computed tomography (CT) of the chest and abdomen confirmed the liver lesions and multiple lesions in the pancreas and lungs, with no significant abdominal or thoracic lymphadenopathy (Figures 1 and 2 ).

Based on the radiologic investigations, the etiology of her CS was strongly suspected to be due to ectopic ACTH secretion from an advanced malignancy of uncertain primary origin.

Before we could complete the workup, the patient developed urosepsis, complicated by septic shock, leading to her unfortunate demise. Her autopsy findings revealed a large tumor in the lower lobe of the left lung (40 × 35 × 25 mm). Numerous tumor deposits were noted in the liver, with smaller deposits seen in the thyroid, heart (endocardium), liver, pancreas, spleen, left kidney, and peritoneal lymph nodes. The brain (pituitary) and adrenal glands were normal. Right kidney pyelonephritis was confirmed as the source of sepsis.

**Figure 3:**
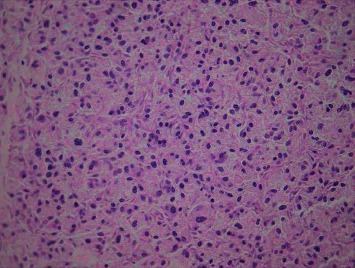
Haematoxylin-and-eosin- (H&E-) stained section of the liver showing a characteristic nesting pattern (“Zellballen”) of cells (400× magnification).

On histology, all the metastatic tumor deposits, including the tumor in the left lung, were similar to the tumor in the liver. The liver section showed a nesting (Zellballen) pattern of cells within a prominent vascular network (Figure 3). The cells were round with granular eosinophilic cytoplasm. Nuclear atypia and mitotic figures were also evident.

**Figure 4:**
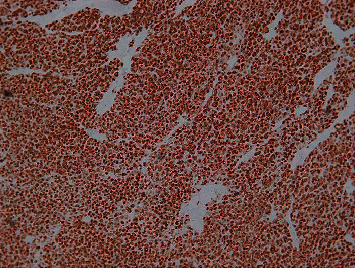
Photomicrograph (100×) of the liver showing strong diffuse staining of tumor cells with a chromogranin.

Immunohistochemistry stained positive for chromogranin (Figure 4) and synaptophysin and negative for cytokeratin 7 and 20. ACTH staining was not shown in both the lung and liver lesions. Immunostaining for CRH is not available in our setting, nor are we able to measure CRH levels in plasma.

Based on the histological and immunohistochemistry findings, a diagnosis of metastatic paraganglioma with paraneoplastic phenomena resulting in CS was made. The cause of death was deemed as systemic sepsis.

## 3. Discussion

Pheochromocytomas (PCCs) and paragangliomas (PGLs) are rare neuroendocrine tumors, collectively termed pheochromocytoma-paraganglioma (PPGL). PGLs are grouped based on their origin in the parasympathetic or the sympathetic nervous system. Sympathetic PGLs are most commonly found in the para-aortic region of the abdomen, pelvis, and chest [3, 4]. Parasympathetic PGLs typically arise from the head and neck region, including the carotid body, vagus nerve, and jugular foramen, of which less than 5% are malignant [5].

The most common sites for PGLs with catecholamine hypersecretion are localized in the abdomen and pelvis. The catecholamine secretion causes paroxysmal episodic hypertension, often accompanied by palpitations, headache and/or hyperhidrosis. Nonfunctional or silent PGLs most commonly manifest as abdominal pain or an incidentally found mass.

25–40% of PPGLs have an underlying germline mutation, of which a mutation in the succinate dehydrogenase (SDH) family is most common. Patients with a mutation in the B subunit of SDH (SDHB) are more likely to have metastatic disease. Other frequent syndrome-associated PPGLs secondary to germline mutations include Von Hippel–Lindau disease (VHL), Multiple Endocrine Neoplasia 2 (MEN2), and Neurofibromatosis type 1 (NF1) [5, 6].

The histological features of PGLs are similar to those of adrenal PCCs. The tumor cells usually show chief cells arranged in nests, alveolar-like, and stereo-like structures surrounded by sustentacular cells partly or entirely with delicate fibrovascular stroma (a Zellballen pattern), although they may also grow in a diffuse pattern [7].

Synaptophysin is an integral membrane protein of small synaptic vesicles in the brain and endocrine cells and is abundant in neuroendocrine cells of diffuse origin and tumor tissues with neuroendocrine function [8, 9]. Chromogranin A is the precursor to several functional peptides, which negatively modulate neuroendocrine function in an autocrine and paracrine manner. It is produced by chromaffin cells of the adrenal medulla, paraganglia, and beta cells of the pancreas and is found to be significantly elevated in serum in patients with neuroendocrine tumors [10]. In addition, it can be assessed on immunohistochemistry in neuroendocrine tissue.

Cytokeratins are keratin proteins found in the intracytoplasmic cytoskeleton of epithelial tissue [11]. They are an important component of intermediate filaments. Immunohistochemistry staining for cytokeratin is helpful in the diagnostic differentiation of metastatic lesions and assists in determining the site of origin [12].

The combination of cytokeratin negativity and chromogranin and synaptophysin positivity on immunohistochemistry is required for a definitive diagnosis of PGLs.

The natural history of PPGLs varies from a benign tumor cured with surgery to the development of rapidly progressive metastatic disease with approximately 10% of PCCs and 25% of PGLs being malignant [4]. The overall 5-year survival rate ranges between 20 and 70% in patients with metastatic disease in comparison to those without metastatic disease whose survival is 90% [13]. Malignancy is defined by the presence of metastatic lesions at sites where neuroendocrine tissue is not normally present, e.g., the lymph nodes, bone, lung, and liver [14].

Our patient's immunohistochemistry stained positive for both chromogranin and synaptophysin and negative for cytokeratin. The combination of Zellballen pattern on histology, a cytokeratin negative stain, and positive chromogranin and synaptophysin staining on immunohistochemistry confirm that our patient had a PGL. On morphological examination, all the tumor deposits in the various organs were identical. The finding of tumor deposit in sites where neuroendocrine tissue is not usually present confirmed the diagnosis of disseminated malignant PGL in our patient.

CS is a rare endocrine condition with an estimated incidence of 2-3 cases per million people per year [15, 16]. CS compromises a group of signs and symptoms that reflects prolonged and inappropriately high exposure of glucocorticoids (endogenous or exogenous) to various tissues. Whilst classical CS is clinically unmistakable, the spectrum of clinical features is broad and nonspecific, often overlapping with a variety of medical conditions including type 2 diabetes mellitus, obesity, and polycystic ovarian syndrome. The unique clinical features of CS include violaceous striae, facial plethora, proximal muscle weakness, easy bruisability, and unexplained osteoporosis [17]. Symptoms are determined by the level of hypercortisolemia and not by the duration of exposure or the size of the tumor [18–21].

Whilst the clinical features of CS may be subtle, the metabolic abnormalities are more upfront. Hypokalemia may be present in up to 58% of cases [22]. Other common metabolic abnormalities include metabolic alkalosis and hyperglycemia.

The exact biological mechanism of ectopic ACTH secretion is unknown [2]. Whilst clinical features of CS due to ectopic ACTH secretion are similar to those due to pituitary ACTH secretion, they tend to have a more rapid onset and progression. Common causes of ectopic ACTH secretion include small-cell lung carcinoma (SCLC), bronchial carcinoid, and neuroendocrine tumor of the lung, small intestines, and the pancreas [23]. Thymic carcinoid and medullary carcinoma of the thyroid may also secrete ACTH, but these are rare [24–26].

Prior to this case, only a few cases of ectopic ACTH secretion associated with PGLs have been described in the literature [27, 28]. As with our patient, in the majority of PGL cases with ectopic ACTH production, patients are female and have metabolic features (hypertension, hyperglycemia, and hypokalemia) [27, 28]. However, very few have shown catecholamine hypersecretion [27]. Most patients with PGL who secrete ACTH have significantly elevated plasma cortisol and ACTH levels [27, 28]. However, there is one case described with a normal plasma ACTH level and another with low ACTH levels [27].

In our case, biochemistry failed to demonstrate excess ACTH in serum. This may suggest that the tumor cells produced a small amount of ACTH, which stimulated the synthesis of cortisol. Excessive cortisol would then inhibit the secretion of ACTH in the pituitary as a negative feedback. A normal ACTH level in the face of an extremely elevated cortisol level is inappropriate and suggests ACTH secretion independent of the control of the hypothalamic-pituitary axis (HPA).

In our patient, postmortem specimens failed to stain for ACTH. This may have been due to the severe necrosis and autolysis of the tumor. Normal pituitary and adrenal glands on autopsy (both macroscopic and histological), elevated serum cortisol, elevated 24-hour urinary free cortisol level and ACTH levels, and failure to suppress on a high-dose dexamethasone suppression test support the diagnosis of ectopic ACTH production. Immunostaining for CRH is not available in our setting, nor are we able to measure CRH levels in plasma.

## 4. Conclusions

We present a 38-year-old female, with widespread metastatic PGL, with CS due to ectopic ACTH secretion. Whilst the diagnosis of CS was confirmed while the patient was alive, the diagnosis of metastatic PGL was made on postmortem as the patient demised whilst still being investigated. Autopsy findings showed multiple tumor deposits with histological examination revealing a Zellballen pattern in keeping with a PGL. Positive immunohistochemistry staining for chromogranin and synaptophysin combined with negative staining for cytokeratin sealed the diagnosis of a PGL. The nondemarcated, widespread autolytic degradation of the tissue is likely responsible for the loss of demonstrable hormone expression (ACTH staining).

It is possible that our patient may have had an underlying genetic mutation predisposing her to develop metastatic PGL; however, due to her demise prior to the diagnosis; we were unable to offer her genetic testing. Her prognosis was poor in view of such widespread disease. The hypersecretion of cortisol may have resulted in hyperglycemia and suppression of her immune system, which predisposed her to severe infections, leading to her demise. Whilst her underling HIV may have contributed, we considered this unlikely due to her undetectable viral load.

This case represents a very rare cause of ectopic CS, most likely caused by an ACTH-producing metastatic PGL.

## Figures and Tables

**Table 1:** Summary of biochemical results.

Variable	Result	Reference range
White cell count	8.45	3.90–12.60 × 109/L
Hemoglobin	**9.0**	11.6–16.4 g/dl
Mean cell volume	89.6	78.9–98.5 fL
Platelet count	311	186–454 × 109/L
Sodium	145	136–145 mmol/L
Potassium	**3.2**	3.5–5.1 mmol/L
Urea	5.6	2.1–7.1 mmol/L
Creatinine	65	49–90 umol/L
eGFR (estimated glomerular filtration rate)	104	>89 mL/mim/1.73 m^2^
HbA1 C (glycated hemoglobin)	**7.6**	<6.5%
Fasting blood glucose	**11.6**	3.3–6.0 mmol/L
Calcium	2.28	2.15–2.50 mmol/L
Magnesium	0.87	0.63–1.05 mmol/L
Inorganic phosphate	0.75	0.78–1.42 mmol/L
ALP (alkaline phosphatase)	58	42–98 U/L
GGT (gamma-glutamyl transferase)	40	<40 U/L
TSH (thyroid-stimulating hormone)	0.84	0.35–5.50 mIU/L
8 am cortisol	**1118**	133–537 nmol/L
ACTH (adrenocorticotropic hormone)	4.6	1.6–13.9 pmol/L
24-hour urinary free cortisol (1^st^ sample)	**10447**	10–124 nmol/24 hours
24-hour urinary free cortisol (2^nd^ sample)	**9856**	10–124 nmol/24 hours
Midnight cortisol	**1042**	<50 nmol/L
Chromogranin A	**750.3**	0.0–84.7 ng/mL

**Table 2:** Low- and high-dose dexamethasone suppression test.

Dexamethasone suppression	1 mg	8 mg
Time of administration	0 : 00	0 : 00
Midnight cortisol	1042	1750
8 am cortisol	796	1253

## Data Availability

No data were used to support this study.

## Abbreviations

PGL: Paraganglioma
PPGL: Pheochromocytoma-paraganglioma
CS: Cushing's syndrome
ACTH: Adrenocorticotropic hormone
CT: Computed tomography.
